# Intersection of Climate and Environmental Change and Priority Health Outcomes Across the Life Course: Working Group 1 Report from the “Agriculture and Diet: Value Added for Nutrition, Translation, and Adaptation in a Global Ecology” (ADVANTAGE) Project

**DOI:** 10.1016/j.advnut.2026.100673

**Published:** 2026-06-11

**Authors:** Kristie L Ebi, Nancy F Krebs, Alexandra L Bellows, Kartik Shankar, Andrew Y Chang, Ronald Kleinman, Daniel J Raiten

**Affiliations:** 1 Department of Global Health, University of Washington Center for Health and the Global Environment, Seattle, WA, United States; 2 Department of Pediatrics, University of Colorado Anschutz—School of Medicine, Aurora, CO, United States; 3 Division of Global Agriculture and Food Systems, The University of Edinburgh, Easter Bush Campus, Midlothian, United Kingdom; 4 Agricultural Research Service, Texas A&M University, College Station, TX, United States; 5 Section of Cardiovascular Medicine, Department of Medicine, Yale School of Medicine, New Haven CT, USA; 6 Department of Pediatrics, Harvard Medical School, Boston, MA, United States; 7 Eunice Kennedy Shriver National Institute of Child Health and Human Development, National Institutes of Health, Bethesda, MD, United States

**Keywords:** climate change, environmental change, health outcomes, life course, nutrition

## Abstract

An ecological approach examining multiple factors, internal (e.g., genetics and concurrent health status) and external (social determinants of diet/nutrition and physical environment), is essential for a comprehensive understanding of the interactions between climate and environmental change (CEC), nutrition, and health. We present evidence on the intersection of CEC and priority health outcomes across the life course. We include examples of observed impacts and future risks of exposure to weather variables (e.g., heat stress, humidity, and air quality) on health outcomes over the life course for particularly vulnerable groups relative to specific physiologic functions. For each life stage, we identify links between climate-related risks, undernutrition and overnutrition, micronutrient status, and health outcomes. From this review, we propose key knowledge gaps and research priorities.

**Statement of Significance**

Working Group 1 of the ADVANTAGE (“Agriculture and Diet: Value Added for Nutrition Translation, Adaptation in a Global Ecology”) Project describes the evidence on the intersection of climate and environmental change and priority health outcomes to inform the assessments in the other Working Groups. This contribution explores the observed impacts and future risks of exposure to weather variables (e.g., heat stress, humidity, air quality, etc.) on health outcomes over the life course for particularly vulnerable groups relative to specific physiologic functions.

## Introduction

The ADVANTAGE (“Agriculture and Diet: Value Added for Nutrition Translation, Adaptation in a Global Ecology”) Project evaluated the evidence base for programs, guidance, and interventions to address nutrition and health challenges in the United States and globally within the context of the potential impacts of climate and environmental changes (CEC) [1]. The ADVANTAGE Project emphasized the role of sustainable nutrition and its ecology (e.g., the interplay of internal and external factors). Interventions focused solely on hunger and food/nutrition insecurity are increasingly insufficient to address the additional challenges for health and nutrition posed by CEC [1].

**FIGURE 1:**
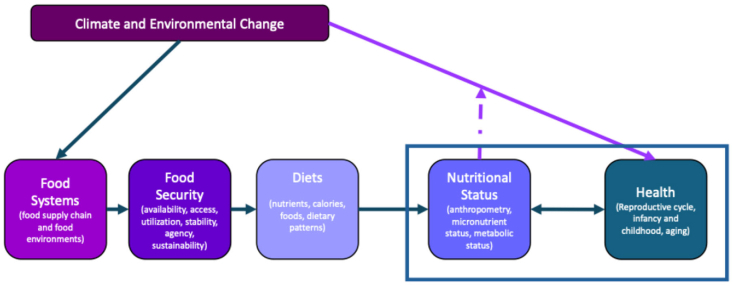
Framework for ADVANTAGE WG 1. Pathway 1: climate and environmental change impacts food systems, resulting in changes to diet and nutritional status that therefore impact health (↔). Pathway 2: climate and environmental change has direct impacts on human health (→) that may be modified by an individual’s status (− • →). ADVANTAGE, Agriculture and Diet: Value Added for Nutrition, Translation, Adaptation in a Global Ecology; WG, working group.

**FIGURE 2:**
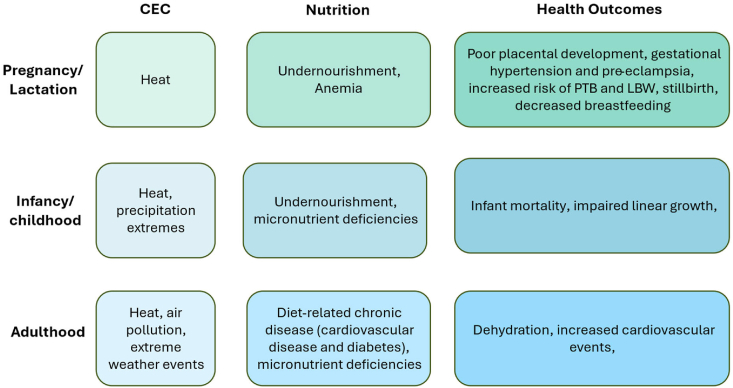
Life stage and outcomes for ADVANTAGE WG 1. ADVANTAGE, Agriculture and Diet: Value Added for Nutrition, Translation, Adaptation in a Global Ecology; CEC, climate and environmental change; LBW, low birth weight; PTB, preterm birth; WG, working group.

This paper reports the results from Working Group (WG) 1 that explored the evidence of the impact of CEC, focusing on climate change, health, and nutritional status using a life course approach, as outlined in FIGURE 1, FIGURE 2.

We synthesize evidence for priority health outcomes (e.g., pregnancy/birth outcomes, noncommunicable diseases, and obesity), providing the foundation for the WGs focusing on dietary patterns and consumer choice (WG2) [2], and on food systems (WG3). WGs 4 and 5 [3] focused on analytical approaches to address complex systems and critical issues in translation and implementation science relevant to the goal of ADVANTAGE.

We first review the risks of climate change for health, followed by a review of evidence linking malnutrition, CEC, and priority health outcomes, including micronutrient deficiencies, overnutrition, and double burden of malnutrition. We then discuss examples of observed impacts and future risks of exposure to weather variables (e.g., heat stress, humidity, air quality, etc.) on nutrition and health outcomes over the life course for particularly vulnerable groups relative to specific physiologic functions. For each life stage, we identify links between climate-related risks, undernutrition and overnutrition, micronutrient status, and health outcomes. Key knowledge gaps and research priorities are outlined before conclusions are synthesized.

## Methods

Given the breadth of the mandate, a narrative literature review focused on evidence linking CEC, malnutrition, and priority health outcomes was undertaken to inform assessments in WGs 2 and 3. For each topic, iterative searches were conducted using PubMed and Web of Science to identify relevant publications. Regular (≥6 times annually) discussions across the author team were used to hone the scope of the review, such that there were ongoing literature searches when topics were refined or added. Further discussions were held across the WGs to facilitate linkage, reduce overlap, and ensure coverage of topics where there was agreement to include. Therefore, there were multiple literature searches across the writing of the narrative review. There was no prespecified search strategy; other than papers were relevant, robust, and informative. Relevance was determined by consensus of the author team. We recognize this approach did not include all possible connections between CEC, malnutrition, and health, such as climate change affecting the geographic range and seasonality of infectious diseases (e.g., malaria and diarrheal diseases) that then impact susceptibility to malnutrition. We also did not include explicit considerations of socioeconomic and political factors.

### Overview of climate change and health

Multiple processes regulate the stability and resilience of the Earth system, including climate change, ocean acidification, stratospheric ozone depletion, phosphorus and nitrogen flows, global freshwater use, land use change, biodiversity loss, atmospheric aerosol loading, and chemical pollution [4]. The Intergovernmental Panel on Climate Change concluded that anthropogenic climate change is already causing excess injuries, illnesses, premature deaths, and adverse mental health outcomes in populations worldwide, and is affecting the ability of health systems to protect and promote human health and well-being [5].

Depending on the level of timely and sufficient investments in health systems and in reducing greenhouse gas emissions, adverse health risks will continue to intensify, many of which can be exacerbated or ameliorated by nutritional status [6,7]. Broadly, these include heat-related illnesses and deaths, illnesses and deaths caused by other extreme weather and climate events (e.g., floods, wildfires, droughts, tropical cyclones, and storm surges), illnesses caused and/or exacerbated by poor air quality (e.g., air pollution and aeroallergens), food and water insecurity and safety, malnutrition in all its forms, and selected infectious diseases [7]. These health impacts have multisystem pathophysiologic effects that permeate the entire life course, with notable risks for women, reproductive outcomes, infants and young children, and the elderly, discussed in further detail in subsequent sections [5]. In general, without adaptation and mitigation, risks will increase with each additional unit of warming [8].

The Intergovernmental Panel on Climate Change synthesized the extent to which current and future generations could experience a hotter and different world depending on choices made now and in the near term to reduce emissions of greenhouse gases [7], showing the observed and projected increases in global mean temperature since 1900. The more rapid temperature increases since 1980 mean that a child born in 2020 will experience very different temperature patterns over their lifetime than those experienced by children born in 1950 or 1980. The extent of that difference in the coming decades will depend on how rapidly greenhouse gas emissions are reduced over the near term. These possible futures need to be considered when assessing the potential consequences of climate change on human health and well-being. Relevant examples of recognized effects of malnutrition and their potential interactions or exacerbations with CEC are presented in the following sections.

### Overview of CEC and malnutrition across the life course

Fully appreciating the intersection of CEC, food systems, diet, nutrition, and health begins with a fundamental understanding of the myriad causes of malnutrition. Malnutrition presents in various forms, including micronutrient deficiencies (which often overlap with other forms of malnutrition), undernutrition (insufficient intake of calories), overnutrition (excessive intake of calories), and vulnerabilities due to physiological perturbations associated with factors such as developmental stage, xenobiotic exposure, and health status [9,10]. Globally, every country experience high levels of ≥1 form of malnutrition, and an increasing number of countries and individuals are experiencing high levels of multiple and coincidental forms of malnutrition [[11], [12], [13]]. The term “syndemic” has aptly been applied to the phenomenon of the co-occurring pandemics of malnutrition in all its forms and climate change [14,15]. Analyses emphasize the shared drivers of these pandemics, the critical need to address them simultaneously, and the potential benefits of doing so [14], as malnutrition affects an individual’s susceptibility to harm from climate-related exposures and recovery from the consequences of these exposures. In the following sections, we outline examples of how CEC may impact nutrition and health across the life course and note gaps in the existing evidence.

### Examples of the impact of CEC on nutrition and health across the life course

The absorption, metabolism, and excretion of nutrients may be adversely affected by the physiologic consequences of CEC. These consequences on human biological systems may include systemic inflammation, increased gastrointestinal permeability with diminished intestinal barrier function, and elevated oxidative stress, each of which can affect nutrient requirements, bioavailability, and status. Full exploration of the myriad interactions between CEC and nutrition is beyond the scope of this review. Instead, illustrative examples are provided, starting with Text Box 1 [[16], [17], [18], [19], [20], [21]] that shows how multifactorial CEC-related interactions can contribute to iron deficiency and possibly anemia (Text Box 1).

Text Box 1 **An illustrative example of how multifactorial climate and environmental change–related interactions can contribute to iron deficiency and possibly anemia**

**Illustrative example**

• There are strong associations between concentrations of air pollution (PM2.5) and anemia prevalence in women of reproductive age [16] and children [17].
  ○ Although the exact biological mechanisms are unclear, PM2.5 or its components contribute to chronic inflammation [18], which can affect iron status. For example, oxidative stress and systemic inflammation coupled with stimulation of hepcidin and inhibition of iron absorption can lead to iron deficiency anemia [19,20].
  ○ Preliminary data also indicate positive associations between ambient temperature and iron deficiency anemia in early pregnancy, with evidence that heat-associated systemic inflammation may further contribute to the risk of iron deficiency and anemia [21].

Alt-text: Text Box 1

The following sections highlight selected life stages with potential susceptibilities to the effects of CEC, especially heat stress. Although data are limited, examples of the interconnections between CEC, nutritional status, and health effects are growing.

### Pregnancy, lactation, infancy, and childhood

#### CEC and pregnancy

Although no population is immune to the adverse effects of CEC, pregnant women and newborns are particularly vulnerable because of their physiology, behaviors, socially determined roles, and susceptibility during humanitarian crises [[22], [23], [24], [25], [26]]. For similar reasons, pregnant women are at an increased risk of undernourishment that can result in maternal mortality, fetal growth restriction, postnatal growth faltering, and an increased risk of obesity in adulthood for the offspring [9].

Robust evidence connects weather variables with adverse maternal and newborn health outcomes [[27], [28], [29], [30]]. Extreme weather and climate events disrupt access to healthcare and increase risks of food and water insecurity and vector-borne illnesses, further amplifying the potential for harm during pregnancy and early childhood [31]. Growing evidence supports associations of heat stress and air pollution with infertility, gestational hypertension and preeclampsia, preterm birth, low birth weight, and stillbirth [32,33].

The biological underpinnings of these findings include the observation that pregnancy is a uniquely vulnerable period for exposure to hot ambient conditions. In animal studies, heat stress in pregnancy impairs placental development and is associated with endothelial dysfunction, inflammation, and oxidative stress, all of which contribute to placental insufficiency [34,35]. Heat stress during human pregnancy is associated with compensatory redistribution of blood flow, which leads to decreased uterine blood flow and altered placental function [30,36]. It also increases the risk of dehydration with counterregulatory hormonal changes, such as antidiuretic hormone and oxytocin release, which can reduce uterine blood flow and alter fetal metabolism [30]. Finally, placental transcriptomic and functional changes are induced [30,[36], [37], [38]].

Maternal malnutrition exacerbates the effects of heat stress. Animal studies support such an interaction with even modest heat stress and marginal dietary restriction, resulting in placental dysfunction and fetal growth restriction [39]. Placentas from undernourished women exposed to heat stress in early pregnancy demonstrated altered expression of hundreds of genes [37]. Histologic evidence of placental malperfusion underlies many of the adverse outcomes associated with infants who are born too small or too soon (collectively referred to as “small vulnerable newborns”) [40].

Heat exposure in pregnancy is directly associated with increased risk of gestational hypertension and preeclampsia. A national cohort of ∼2 million pregnant women in China found an increased risk of both conditions with exposure to higher temperatures in the first half of pregnancy, but with possible protection from these effects with higher temperature exposure in the preconception period [41]. A study from South Africa reported that the largest association between ambient temperature and gestational hypertensive disease occurred in the first 3 to 4 wk of pregnancy, consistent with the effect of heat on early placental development [42].

Associations have been reported between temperature maximums across pregnancy and obstetric and neonatal outcomes in South Asia [43]. Data collected between 2014 and 2020 from over 126,000 pregnant individuals across 3 South Asian sites who participated in a maternal–neonatal birth registry [44,45] were linked with meteorological records collected at regional weather stations. Exposure to maximum temperatures in the first and second trimesters were associated with increased risk of preterm birth and low birth weight, and third-trimester heat exposure was associated with a greater incidence of preeclampsia. No trimester was without risk, and adverse outcomes with greater temperatures varied depending on timing during gestation and the geographic location [43].

Studies of preterm births conducted in high-income countries quantified the effects of higher ambient temperatures. An analysis from Northern California indicated an 11.6% increase in preterm births per 5.6°C increase in average weekly mean heat index [46]. An analysis of medical records from 12 United States sites that included 223,375 singleton deliveries also reported that acute and chronic ambient temperature extremes increased the risk of preterm birth [47].

A systematic review of 70 studies primarily from high-resource settings reported the impact of high temperatures on the risk of preterm birth, low birth weight, and stillbirth [32]. A meta-analysis of 6 studies evaluating preterm birth estimated a 16% higher risk on heatwave days compared with nonheatwave days and a 1.05-fold increase in the odds of preterm birth for each 1°C increase in temperature throughout pregnancy. In that review, only a quarter of the included studies originated in low- and middle-income countries (LMICs). A subsequent, larger systematic review and meta-analysis, including ∼198 studies from 66 countries (37% LMIC), confirmed, with very high certainty, similar increased odds of preterm birth with increasing temperature, and a 1.25-fold increase in odds during heat waves. Other outcomes associated with high certainty were increased odds of hypertensive disorders of pregnancy, gestational diabetes, stillbirths, and neonatal admissions [48].

Despite this growing recognition in LMICs, where the risk of adverse effects is higher due to endemic maternal malnutrition, including underweight, overweight, and micronutrient deficiencies, nutritional status remains an unmeasured variable in most studies examining health impacts associated with climate variability. A prospective cohort study of heat exposure in undernourished pregnant women (average BMI 22) in southern India engaged in moderate-to-heavy physical work documented 3.5 times greater (compared with unexposed participants) self-reported heat stress symptoms (e.g., headache, fatigue, skin rashes, and urogenital symptoms); 50% higher likelihood of demonstrating physiologic strain (e.g., elevated core body temperature and elevated urine specific gravity) that was higher at end of the work shift; and a 2-fold increased risk of adverse fetal/maternal outcomes [49]. In a secondary analysis of a randomized trial, lower birth length and head circumference were associated with maximal daily temperatures in the first trimester in Pakistani women with chronic malnutrition and micronutrient deficiencies [37]. The associations were mitigated for women randomly assigned to comprehensive nutritional supplementation, including energy, protein, and multiple micronutrients, initiated before conception and continued through the entire pregnancy. More granular information on the impacts of high heat during pregnancy and the interaction with maternal nutritional status in LMICs is urgently needed to better understand the magnitude of risks.

#### CEC and lactation

Lactation biology, which includes the range of factors affecting human milk composition, along with infant feeding practices, has profound impacts on early infant postnatal and child growth and development. Examination of these critical components of the infant and child’s exposures requires an ecological approach [50], including the potential for heat exposure during pregnancy to adversely impact both the physiologic preparation for lactation and lactation itself. Although a large body of evidence links ambient heat stress to impaired lactation in agricultural animals [51,52], few studies have examined impacts in lactating humans. At the time of publication, no studies have systematically evaluated the effects of high ambient temperatures on human milk production and composition. A cross-sectional study assessing the impacts of exposure to forest fire smoke during lactation found detectable levels of polycyclic aromatic hydrocarbons in the breast milk of exposed women [53].

Breastfeeding mothers from Burkina Faso were reported to have reduced breastfeeding duration with increasing temperature [54]. Observational studies in China [55] and Bolivia [56] found that less than one-third of lactating (or pregnant) women met recommendations for daily water intake (2–3 L/d), and that lactation status in a rural, hot-humid area was associated with >3-fold greater odds of hypohydration. The dual biologic stresses of high fluid output in milk and insensible losses associated with high ambient temperatures plausibly increase the risk for lactation insufficiency or failure, with consequent issues for infant growth and health. Climate-associated migration adversely affects the support of social and health systems for breastfeeding women, including limited safe spaces to breastfeed, decreased availability of donated breastmilk substitutes, and fragile water and food supplies to support the high energetic, metabolic, and nutritional demands of lactation [57].

The impacts of climate-related exposures on human lactation, milk composition, and growth of the young nursing infant remain exceptionally understudied.

#### CEC and health outcomes in infants and children

In 2024 globally, an estimated 23.2% of children aged <5 y are stunted, which indicates chronic malnourishment, whereas 6.6% experience acute malnourishment in the form of wasting [58]. Most children experiencing malnutrition reside in regions expected to be at higher risk from CEC [7]. Infants have multiple physiologic characteristics**―**many related to their nutritional status―that increase their vulnerability to the adverse effects of CEC, such as high ambient temperatures. Specifically, impaired fluid and nutritional intakes result in an elevated risk for rapid decompensation, including dehydration and electrolyte imbalance, due to immature organ function, a high metabolic rate and high energy and nutrient requirements, rapid growth and limited energy reserves, a greater body surface area in relation to weight in infants compared with adults, and relatively less robust thermoregulation. Young infants are uniquely reliant on a single food (ideally breastmilk) and on the capacity of a caregiver to provide food and shelter. A recent systematic review found that exposures to higher temperatures were associated with an increased risk of infant mortality, hospital admissions, and skin rashes, including those caused by hand, foot, and mouth disease [59]. The strength of other associations was limited by several factors: *1*) heterogeneity in exposure and outcome assessments, *2*) insufficient data from low-resource settings and LMICs, and *3*) a lack of focus on vulnerable subgroups such as those born prematurely and/or with fetal growth restriction.

As highlighted in a technical report from the American Academy of Pediatrics, CEC impacts child health through multiple pathways, including air pollution, heat exposure, natural disasters, food insecurity and nutrition, the changing epidemiology of infections, and mental health harms [60]. The plausibility of interactions among well-recognized socioeconomic drivers of growth impairment and environmental factors was supported in an examination of growth in Pakistani children aged <2 y. Combining household nutrition survey data and ecological datasets, the analysis found potentially independent negative associations among stunting and wasting with indicators of CEC, including heat and excess precipitation [61]. Examples are given in Text Box 2 [[62], [63], [64], [65]], and impacts are discussed in the “Knowledge gaps and research priorities” section.

Text Box 2 **Examples of CEC impacts on priority child health targets**

**Examples of CEC impacts on priority child health targets**

• **Heat stress and stunting:** analyses using the nationally representative Ethiopian Demographic Health Survey reported associations between higher temperatures in the first and third trimesters of pregnancy and greater risk of severe child stunting [62] or wasting [63].
• **Precipitation:** a global analysis of precipitation patterns from 53 countries reported that precipitation extremes, especially drought, were related to impaired child linear growth [64].
• **Temperature and/or precipitation**: recent analyses of 220,000 children in 4 South Asian countries (India, Bangladesh, Nepal, and Pakistan) showed that child growth was undermined by uncommonly wet and dry days, as well as by extreme temperatures in the first 2 y of life [65].

Alt-text: Text Box 2

Abbreviation: CEC, climate and environmental change.

### CEC and adults, including the elderly

The middle and older ages of the human lifecycle―defined as ages 40 to 60 y and >60 y, respectively―are associated with a myriad of changes at the molecular, cellular, and tissue level that increase susceptibility to critical chronic and degenerative diseases [[66], [67], [68]]. Such underlying biological processes include genomic instability, telomere loss, epigenetic changes, chronic inflammation and immune dysfunction, cellular senescence, loss of proteostasis, mitochondrial dysfunction, altered intercellular communication, and exhaustion of stem cell reserves [67]. As such, middle-aged and older adults experience a high incidence of cardiovascular disease, cancer, chronic obstructive pulmonary disease, dementia, and osteoarthritis. The absence of these conditions defines healthy aging during these life stages.

CEC can modify the relationship between aging and chronic disease, as the physiologic changes with aging increase vulnerability to climate impact drivers [[69], [70], [71], [72], [73], [74], [75], [76]]. For example, within the cardiovascular system, aging leads to increased myocardial stiffness, precipitating diastolic dysfunction and impaired ventricular filling [77,78]. Blood vessels lose elasticity, whereas natural pacemaker cells become less responsive to autonomic signals to raise the heart rate [77]. Thus, older adults are less able to increase cardiac output to meet the demands of environmental stressors, such as exposure to high ambient temperatures, and are at higher risk of circulatory collapse from such insults. Chronic poor diet, including diets high in sodium, processed sugars, and saturated fats, can accelerate these pathophysiologic processes via hypertension, dyslipidemia, and diabetes mellitus. This results in premature atherosclerosis and myocardial dysfunction, further impairing the cardiovascular response to environmental stress [79].

Chronic and acute elevations in ambient particulate matter and gaseous fractions of air pollution have increased with climate change for a variety of reasons, including stagnating global wind patterns and fiercer, more frequent regional wildfire events [80,81]. Other climate-related phenomena, such as hurricanes, flooding, tropical cyclones, and winter storms/blizzards, also disproportionately harm the health of older adults. For example, there are notable increases in myocardial infarctions after major tropical cyclones and snowstorms, with health effects sometimes persisting for years [[82], [83], [84], [85]]. In addition, sea level rise may lead to groundwater encroachment of seawater in coastal regions, leading to increases in the sodium content of drinking water [86]. Preliminary analyses suggest that this may have adverse effects on population-level blood pressure trends and hypertension [87,88]. Text Box 3 [70,71,73,74,78,89,90] highlights some of the mechanisms associated with 2 primary CEC-related exposures, air pollution, and heat stress.

Text Box 3 **Mechanisms associated with the impacts of CEC-related exposures on elderly individuals**

**Mechanisms associated with the impacts of CEC-related exposures on older adults**

**Air pollution**

• The aging process reduces pulmonary vascular barrier functions, rendering older adults more susceptible to inhaled toxins [70].
• Half of the 7 million global deaths attributed to air pollution are in individuals aged >65 y, with half attributable to cardiovascular disease and stroke [89].
• Both short-term and long-term air pollution exposure are linked to other disorders of aging, including chronic obstructive pulmonary disease, myocardial infarctions, heart failure, and cardiac arrest [78,90].
• Long-term exposure to air pollution may also be associated with malignancy (particularly lung cancer), dementia, osteoporosis, and frailty [71,90].

**CEC-specific insults (heat stress)**

• Extreme heat and heat waves primarily claim the lives of people aged >65 y, predominantly through cardiovascular disorders.
• Advancing age is associated with reduced capacity to mobilize splanchnic blood flow from the gut and kidneys to the surface vessels [73,74].
• The less compliant older heart is less compliant and thus at increasing cardiac output in response to thermal stress (or does so at the expense of myocardial ischemia). Elevated serum troponins (markers of myocardial ischemia and injury) are an independent risk factor for poor outcomes from heat injury [78].
• Older adults with pre-existing renal or cardiovascular diseases are frequently prescribed medications such as diuretics and beta blockers that induce dehydration or blunt the ability of the heart to augment its output and heart rate [76].
• The aging process leads to a decrease in skin thermoreceptor density and sweat gland localization near the body’s core, which together impair the innate evaporative cooling mechanisms of the body [72,75].

Alt-text: Text Box 3

Abbreviation: CEC, climate and environmental change.

In addition to these pathophysiological susceptibilities, there are socioeconomic factors unique to older adults that further exacerbate associations between CEC, aging, and chronic diseases. The health risks posed by some climate impact drivers, such as wildfires and heatwaves, can be reduced using personal protective equipment such as air filters and air conditioning units. Older adults face financial vulnerabilities, such as living on fixed incomes, which limit their ability to invest in such equipment. They are less likely to live in air-conditioned homes, and even elders whose residences have air conditioning are less likely to use them on hot days [91,92].

This is particularly threatening given that the aging process leads to a reduction in thermoreceptors that engage the autoregulatory mechanisms of the body [75]. Relocation is also more challenging for older people because many lacks the physical mobility and transportation resources to move away from CEC-mediated hazards [[93], [94], [95], [96]]. Elders who reside in nursing homes and other institutionalized settings are particularly constrained in their ability to quickly relocate. That said, even independently living older adults are less likely to be able to drive, are more likely to live by themselves, and are less able (and sometimes less willing) to relocate to a safer location during natural disasters [93].

These financial and mobility challenges also may lead to unfavorable dietary choices among elders, leading to a preference for prepared or processed foods that are high in sodium and deleterious lipids/processed sugars, and low in fresh vegetables and fiber [97]. Furthermore, social isolation, depression, and cognitive decline among older adults exacerbate malnutrition, which is a major risk factor for multiple chronic diseases, including cardiovascular disorders [98].

### Summary of CEC impacts over the life course

Pregnancy, lactation, infancy, and childhood represent critical periods of vulnerability due to their dynamic and intense physiologic stressors, which include nutritional stress. Older adults present with different but equally important pathophysiological susceptibilities. Emerging evidence demonstrates that various aspects of a changing environment, including extreme heat and precipitation, are having profound impacts on health across the life course. Moreover, the threats imposed by CEC are heightened by the distinct risks for poor nutrition at all life stages. Our ability to ascertain the true risk associated with the nexus of CEC, food, nutrition, and health demands a deeper appreciation of these complex relationships.

### Knowledge gaps and research priorities

There are many knowledge and data gaps in our understanding of the interactions of CEC with nutrition-related health conditions across the life course [99].

Current approaches to public health surveillance (e.g., NHANES and Department of Human Services surveys) do not consider the impact of CEC, which severely limits their ability to meaningfully describe risks. The lack of consideration of the potential impact of CEC on an individual’s nutritional status makes a compelling case for the value of the ecological approach. Methods that are potentially useful for actualizing this approach (e.g., measures, metrics, and analytical frameworks) are covered in WG 4.

It is unclear how overnutrition and undernutrition influences human vulnerability or resilience to mechanisms underlying climate health impacts. Micronutrient requirements may be higher in vulnerable groups due to reduced bioavailability, increased demand, and/or greater losses. It is critical to investigate how specific tissues and biological systems are impacted by CEC and nutrition, e.g., barrier functions in the pulmonary vasculature (i.e., the interface for air pollution and the systemic circulation); the integrity and function of the gastrointestinal tract; the physiologic responses of the cardiovascular system to extreme heat; cognition and neurodegeneration; tumorigenesis (particularly, highly environmentally associated malignancies such as lung cancer); sensory functions (e.g., hearing and vision loss); bone health (osteoporosis, osteoarthritis, and major fractures); the immune system, systemic inflammation, and immunoaging; and the effects of specific ingested toxins (e.g., heavy metals and endocrine disruptors).

In conclusion, because of their reciprocal nature, the impact of climate on health cannot be assessed without consideration of nutrient intake and utilization, and vice versa. Although specific vulnerabilities predominate at different points in the life course, common physiological effects of CEC and of dietary and nutritional insufficiency combine to impact the health and resilience of populations and individuals. Addressing the complexity of these interactions is an urgent research challenge.

## Author contributions

The authors’ responsibilities were as follows — KLE, NFK, DJR: conceptualized the topic and content framework; KLE, NJK, ALB, KS, AYC, RK: reviewed and synthesized information for inclusion in the initial and subsequent drafts; and all authors: wrote and edited the review and read and approved the manuscript.

## Funding

The authors reported no funding received for this study.

## Declaration of generative AI and AI-assisted technologies in the writing process

The authors declare that no generative AI or AI-assisted technologies were used in the writing of this manuscript.

## Conflict of interest

The authors report no conflicts of interest. The contents of this article represent the authors’ views and do not constitute an official position of the National Institutes of Health or the United States Government. NFK is on the Editorial Board for the *American Journal of Clinical Nutrition*.
